# Human Health Risk Assessment Based on Toxicity Characteristic Leaching Procedure and Simple Bioaccessibility Extraction Test of Toxic Metals in Urban Street Dust of Tianjin, China

**DOI:** 10.1371/journal.pone.0092459

**Published:** 2014-03-20

**Authors:** Binbin Yu, Yu Wang, Qixing Zhou

**Affiliations:** 1 Key Laboratory of Pollution Processes and Environmental Criteria (Ministry of Education), College of Environmental Science and Engineering, Nankai University, Tianjin, China; 2 Department of Agricultural and Biological Engineering, University of Florida, Gainesville, Florida, United States of America; The Ohio State University, United States of America

## Abstract

The potential ecological and human health risk related with urban street dust from urban areas of Tianjin, China was quantitatively analyzed using the method of toxicity characteristic leaching procedure (TCLP) and simple bioaccessibility extraction test (SBET). In the study, Hakason index, Nemerow index (*P*), the hazard index (*HI*) and the cancer risk index (*RI*) were calculated to assess the potential risk. The sequence of potential ecological risk based on Hakason index was arsenic (As) > cadmium (Cd) > lead (Pb) > copper (Cu) > chromium (Cr), in particular, As and Cd were regarded as high polluted metals. While the results of extraction of TCLP were assessed using *P*, the sequence was As > Pb > Cd > Cr > Cu, which mean that As and Pb should be low polluted, and Cd, Cr and Cu would barely not polluted. For human health, total carcinogenic risk for children and adults was 2.01×10^−3^ and 1.05×10^−3^, respectively. This could be considered to be intolerable in urban street dust exposure. The sequence in the hazard quotient (*HQ*) of each element was As > Cr > Pb > Cu > Cd. The *HI* value of these toxic metals in urban street dust for children and adults was 5.88×10^−1^ and 2.80×10^−1^, respectively. According to the characters of chemistry, mobility, and bioavailability of metals in urban street dust, we estimated the hazards on the environment and human health, which will help us to get more reasonable information for risk management of metals in urban environment.

## Introduction

Along with rapid urbanization, many suburban lands are being converted to residential use, streets, commercial and industrial zones. The high population density leads to an increasing level of urban environmental pollution, industrial discharges, traffic emissions, and waste from municipal activities cause the major anthropogenic troubles [1]–[4]. Street dust is an important factor of urban pollution, and road activities often cause air pollution and adverse human health effects such as lung cancer, hypertension and cardiovascular diseases [5]–[9]. Due to the accumulation of metals in street dust with atmospheric deposition by sedimentation interception, human health may be adversely affected by air pollution if metal concentrations reach a level of being considered as toxic pollutants [10].

Many studies reported that the total concentration of heavy metals in street dust was regarded as an indicator of urban air pollution affecting urban environmental quality. More air-environmental scientists described that not only the total concentration of heavy metals in street dust, but also the proportion of their mobile and bioavailable forms were important to environmental and human health risk of metal pollutants [11]–[16]. Metals in urban street dust occur in variable forms, such as adsorbed or exchangeable. The forms of toxic metals could affect their mobility and bioavailability including uptake by living organisms, and result in potential risk to the environment and residents. To evaluate long-term impacts of toxic metals on urban environment and risks to residents, it was necessary to investigate chemical forms, mobility, and bioavailability of toxic metals in urban street dust.

Tianjin (39°07′ N, 117°12′ E) is a mega-city in northern China, which is adjacent to Beijing and Hebei Province. It is a municipality along the coast of the Bohai Gulf. There are four distinct seasons, characterized by cold, windy, dry winters affected by the vast Siberian anticyclone, and hot, humid summers due to the East Asian monsoon. The mean temperature is 11.6–13.9°C and the annual average wind speed is 2–5 m s^−1^. Rapid expansion in Tianjin has made the city become one of the most densely populated regions in China, with the population size of over 1.27 million. Urbanization increases the population density and lots of human activities such as traffic, industry, commerce, petrol combustion, and waste disposal. Of the contaminants in the urban area, toxic metals have caused serious concern of both researchers and governments for their characteristics of accumulation and degradation-resistant. As a crucial component of urban ecosystems, urban dust is subjected to continuous accumulation of contaminants [17]–[19], especially toxic trace metals [9], [20], lots of investigations have been done on metal contamination in urban areas [15], [21]–[25].

In this study, we provided valuable information about toxic metals in urban dust, including chemical characters, mobility, and bioavailability of toxic metals in urban environment. According to the information, we estimated the hazards of toxic metals in street dust, in particular, adverse effects on the environment and human health. The key scientific problems related to street dust pollution were addressed as follows: (1) the spatial distribution of toxic metals in street dust; (2) the mobility and bioavailability of toxic metals; (3) the risk index (RI) and Nemerow index (*P*) based on chemical behaviors and mobility of toxic metals; (4) the hazards index and total cancer risk based on oral bioavailability of toxic metals; and (5) some environmental management advice to reduce dust pollution.

## Materials and Methods

### Sampling (No specific permits were required for this study)

**Figure 1:**
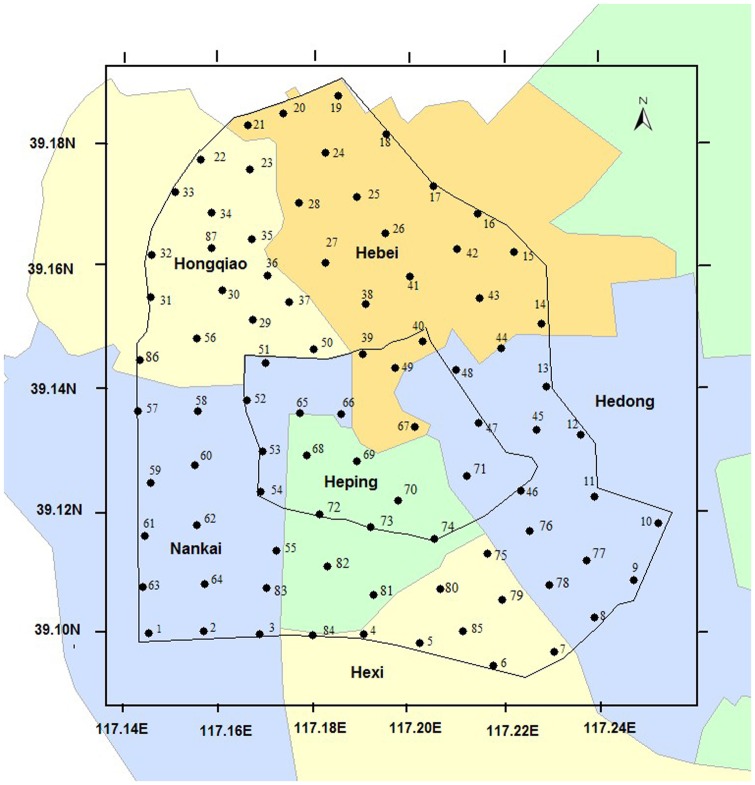
The sampling sites of urban street dust in Tianjin, China.

Urban street dust samples were collected on pavements next to main roads at periods when no rain had occurred during the previous week, brushes and trays were used for sampling. Along the central line in Tianjin, we collected 87 samples (39°09′ N to 39°22′ N, 117°14′ E to 117°27′ E). To uniform samples, the studying area was divided into regular grids of 1×1 km^2^ and avoiding serious polluted areas (such as hospitals, gas stations, and bus stations). Each sample was at least 200 g and composed of 5–7 subsamples. Collected samples were put into clean polythene bags and taken to the laboratory as soon as possible. Each urban street dust sample was thoroughly mixed, sieved through a 63 μm nylon sieve, then one part was air-dried, other part was stored at 4°C. The detailed sampling information was depicted in Fig. 1.

### Sample analyses

The pH values of urban street dust samples were measured with Milli Q water with a solid-to-solution ratio of 1∶2.5 (w/v), organic matter (OM) contents were measured by the wet digestion method, the metals were measured by the microwave assisted acid digestion (US EPA 1996). 0.2 gram of the street dust sample was mixed with 8 mL 65% nitric acid, 5 mL 30% hydrogen peroxide and 3 mL 30% hydrofluoric acid and put into a microwave oven. The digestion procedure was described as follows: stage-1 (10 min to reach 150°C), stage-2 (10 min to reach 180°C) and stage-3 (15 min at 200°C). After cooling below 100°C, sample digestion solutions were evaporated near to dryness, added to 10 mL Milli Q water. Solutions were stored in 10 mL polyethylene vials at 4°C till analysis. Blank and control samples were used to check the accuracy and quality. The contents of arsenic (As), lead (Pb), cadmium (Cd), copper (Cu) and chromium (Cr) were measured by the inductively coupled plasma spectrometry (ICP-MS, DRC-e), the recovery of the metallic elements was in the 85.1–106.8% range.

### Toxicity characteristic leaching procedure (TCLP)

Leaching of hazardous metals from street dust samples were examined by means of a TCLP [26]. The pH values of dust samples were beyond 5, the extraction solution (HOAC, pH = 2.88±0.05) was used, the solid - to - liquid ratio was 1∶20, the temperature was 23±2°C, and the agitation time was 18 h in a rotary tumbler. After extraction, the leaching solutions were filtered through a glass fiber filter (0.45 μm). The filtrates were analyzed by ICP-MS immediately.

### Simple bioaccessibility extraction test (SBET)

Oral bioavailability of metals in street dust samples were measured by SBET [27]–[29]. Samples were extracted with glycine (0.4 M; pH = 1.5±0.05, pre-adjusted with concentrated hydrochloric acid), the solid-to-liquid ratio was 1∶100. Samples were extracted by rotating the samples end-over-end at 37°C for 1 h. The mixture solutions were centrifuged and the supernatant was filtered through 0.45 μm cellulose acetate filter. The pH values of the mixed-solution should be within 0.5 pH units of the starting pH, otherwise the procedure has to be redone. The filtrates were stored in a refrigerator at 4°C until analyzed by ICP-MS in one week.

### Pollution risk of total concentrations of urban street dust

Most researchers used potential ecological risk index (*RI*) as a method to assess the degree of metals pollution in soil. The method was originally introduced by Hakanson [30], according to the toxicity of heavy metals, assessed the response of metals to the environment.
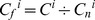
(1)

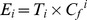
(2)

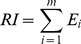
(3)


Where 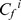 is the single metal pollution factor, *C^i^* is the concentration of a metal in urban street dust, 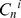 is a reference value for a metal, in this study, which is the soil background value in Tianjin [31]. *E_i_* is the monomial potential ecological risk factor, T_i_ is the response coefficient for the toxicity of the single heavy metal, which for Cd is 30, As is 10, Pb and Cu are 5, Cr is 2 [30], *RI* is calculated as the sum of all five risk factors for heavy metals in urban street dust. Different categories of metal pollution about *E_i_* and *RI* were delineated in Table 1.

**Table 1:** Potential ecological risk categories based on *E*_i_ and *RI* values^a^.

E_i_	Ecological risk categories of single metal	RI value	Ecological risk categories of the environment
E_i_<40	Low contamination	RI<150	Low risk
40≤ E_i_<80	Moderate risk	150≤RI<300	Moderate risk
80≤E_i_<160	Considerable risk	300≤RI<600	Considerable risk
160≤E_i_ <320	High risk	RI≥600	Serious risk
E_i_≥320	Serious risk		

a [30].

### Assessment of pollution risk using the results of extraction of TCLP

To evaluate the toxicity of the mobile and soluble parts of urban street dust, via a TCLP test, Nemerow index (*P*) [32] was used to assess the degrees of dust contamination. The pollution index was defined as the ratio of metal concentration to geometric means of background concentration of the corresponding metal:
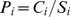
(4)and
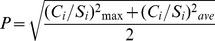
(5)


Where *P_i_* is the evaluation score corresponding to each sample, *C_i_* is the value of extraction of TCLP, *S_i_* is the maximum concentration (mg·L^−1^) of a contaminant for toxicity characteristic, As for 5.0, Pb for 5, Cd for 1.0, Cu for 15, and Cr for 5 [33], *P* is the metal integrated pollution index, (C_i_/S_i_)_max_ is the maximum and (C_i_/S_i_)_ave_ is the average. There are five categories of metal pollution about *P* were displayed in Table 2.

**Table 2:** Potential ecological risk categories based on the *P* values^a^.

P value	Ecological risk category
P≤0.7	Safe
0.7<P≤1.0	Warily level
1.0<P≤2.0	Low contamination
2.0<P≤3.0	Moderate contamination
P>3.0	Serious contamination

a [31].

### Potential human health risk of metals in urban street dust

In order to assess both non-carcinogenic and carcinogenic risk for children and adults from ingesting urban street dust, the chronic daily intakes (CDI) of toxic metals and potential risks were used. According to the Exposure Factors Handbook [34], CDI (mg·kg^−1^·day^−1^) of toxic metals via dust can be calculated using the following equation:
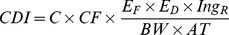
(6)


Where C is the exposure site concentration (mg·kg^−1^), in this study, C is the mean concentration of SBET; E_F_ is the exposure frequency of 350 days·year^−1^ [35]–[36]; E_D_ is the exposure duration, in this study, 6 years for children and 24 years for adults; *Ing*_R_ is the ingestion rate at 200 mg·day^−1^ for children and 100 mg·day^−1^ for adults [34], [37]; BW is average body weight, 15 kg for children and 60 kg for adults [35], [38], and AT is average time (for non-carcinogens, AT = E_D_×365 days; for carcinogens, AT = 70×365 days); CF is conversion factor, 1×10^−6^ kg·mg^−1^.

The potential health risk of each element was calculated with equations (7–8). While Eq.7 is for non-carcinogenic risk, Eq.8 is for carcinogenic risk. 
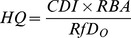
(7)and
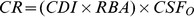
(8)


Where oral reference dose (RfD_o_) and cancer slope factor (CSF_o_) were obtained from regional screening levels [39], relative bioavailability (RBA) is the ratio of SBET/total contents. RfD_o_ for Pb has not established in US EPA, in this study, RfD_o_ for Pb is 3.5×10^−3^ mg·kg^−1^·day^−1^ calculated from the provisional tolerable weekly Pb intake limit (25 μg·kg^−1^-body weight) recommended by FAO/WHO for adults [24], [40]. The toxicity of Cr depends on its valence state (Cr^6+^ and Cr^3+^), in this study, Cr^6+^ represented total Cr. The RBA, RfD_o_ and CSF_o_ values were listed in Table 3.

**Table 3:** Health risk from heavy metals in urban street dust (n = 87).

Contaminant	*C* a (95% UCL) mg·kg^−1^	*RfD_o_* b (mg/kg-day)	*CSF_o_* b (mg/kg-day) ^−1^	Chemical daily intake (mg/kg-day)		Child		Adult	
				Children Adult		HQ	Carcinogenic risk	HQ	Carcinogenic risk
As noncanc.	101.41	3.00E-04		1.35E-03	6.76E-04	4.56E-01		2.28E-01	
As canc.	101.41		1.50E+00	1.11E-04	5.56E-05		1.69E-05		8.44E-06
Cr noncanc.	71.85	3.00E-03		9.58E-04	4.79E-04	6.60E-02		3.30E-02	
Cr canc.	71.85		5.00E-01	7.87E-05	3.94E-05		8.14E-06		4.07E-06
Cd (diet)	0.45	1.00E-03		6.00E-06	3.00E-06	1.33E-03		6.67E-04	
Cu	55.47	4.00E-02		7.40E-04	3.70E-04	3.22E-03		1.61E-03	
Pb^c^	60.11	3.50E-03		8.01E-04	4.01E-04	3.25E-02		1.63E-02	

a 95% upper confidence limit of the mean concentrations.

b [39].

c [40], [24].

Though interactions between some metals might result in their synergistic manner, all metal risks were additive. The hazard index (*HI*) and total risk [41] were estimated with the following equations:
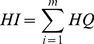
(9)and
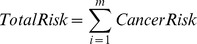
(10)


If *HI* exceeds 1.0, there is a chance that non-carcinogenic effects may occur, with a probability which increases with an increase in the value of *HI*; and then, if *HI* is less than 1.0, it believed that there is no significant risk of non-carcinogenic effects [42]. Cancer risks (*CR*) estimates the incremental individual lifetime cancer risk for simultaneous exposure to several carcinogens, the acceptable or tolerable risk for regulatory purposes is in the range of 1×10^−6^–1×10^−4^.

## Results

### Dust properties and metals spatial distribution

**Figure 2:**
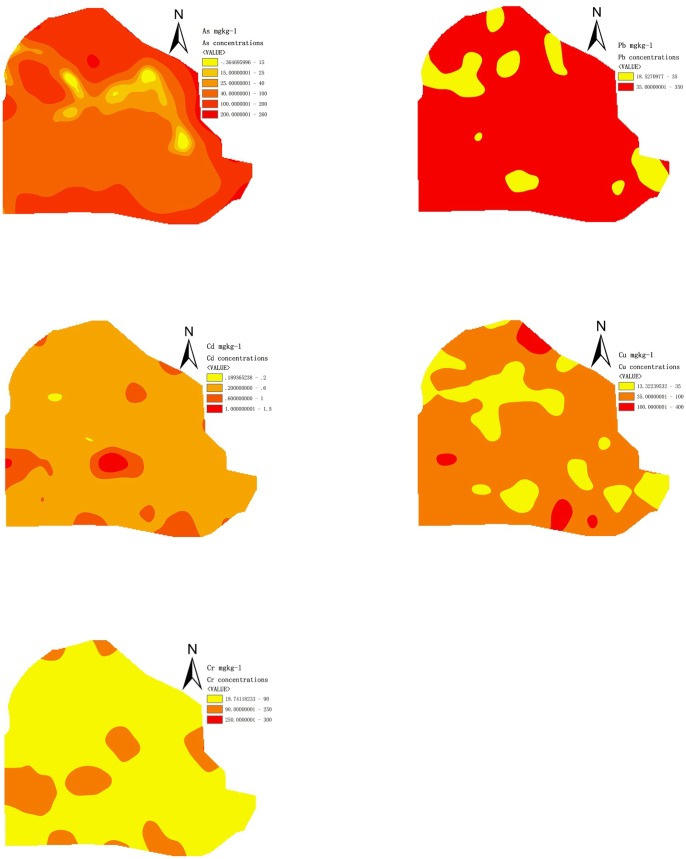
Spatial distribution of metals (As, Pb, Cd, Cu and Cr) in urban street dust of Tianjin, China.

The urban street dust samples from Tianjin cover a wide range of pH and OM. The pH values varied from 7.50 to 10.77 with average of 8.28, OM from 0.56% to 4.25% with average of 2.32%. The concentrations of As, Pb, Cd, Cu and Cr in urban street dust varied from 17.18 to 203.78, 20.64 to 155.67, 0.22 to 1.38, 20.65 to 187.78, and 30.85 to 224.60 mg·kg^−1^, respectively, with the average concentration of 101.41, 60.11, 0.45, 55.47 and 71.85 mg·kg^−1^, respectively (Table 4). The sequence in the contents of toxic metals in urban street dust was As > Cr > Pb > Cu > Cd. The spatial distributions of metals (As, Pb, Cd, Cu and Cr) in urban areas of Tianjin were depicted in Fig. 2. As was strong contaminated in most sites of the urban areas, which was higher than Class III of National Soil Standards [43]. Pb and Cr were lower than Class II of National Soil Standards, while Cd and Cu were lower than Class II of National Soil Standards in most urban areas and only a few of sites were higher than Class III of National Soil Standards where near the sites were high population density, including stations, commercial zones and construction fields.

**Table 4:** A comparison of metal average concentration (mg kg^−1^) in street dust from some cities.

City (Ref.)	As	Pb	Cd	Cr	Cu
Tianjin	101.41	61.11	0.45	71.85	55.47
Beijing^a^	-	61	1.2	85.6	42
Shanghai^b^	8.01	236.62	0.97	264.32	257.63
Shenyang^c^	-	75.29	0.42	-	51.26
Nanjing^d^	13.4	103	1.10	126	123
Xi'an^e^	10.62	230.52	-	167.28	94.98
Hongkong^f^	66.8	120	-	124	110
National Standard- Class I^g^	15	35	0.20	90	35
National Standard- Class II^h^	25	350	0.60	250	100
National Standard- Class III^i^	40	500	1.0	300	400

a [22].

b [21].

c [44].

d [24].

e [50].

f [51].

g [43] Environmental quality standard for soils in China (National Environmental Protection Agency of China, 1995), and values in Class I are threshold levels of nationwide natural background.

h [43] Values in Class II are threshold values established to protect agricultural production and maintain human health.

i [43] Values in Class III are estalbished to maintain normal growth of plants, particularly the trees.

### Metal mobility and relationship with dust properties

The mobility of toxic metals depends on their chemical forms. In this study, according to the method of TCLP extraction, the range of extraction efficiency of toxic metals varied widely among urban street dust, As is 0.24% to 21.22%, Pb is 0.04% to 26.73%, Cd is 0.24% to 75.35%, Cr is 0.54% to 5.64% and Cu is 0.15% to 7.12%. The sequence of average extraction efficiency of toxic metals is Cd (12.99%) > Pb (4.11%) > Cr (2.70%) > As (2.07%) > Cu (1.40%).

The mobility of toxic metals in urban street dust may be affected by metal-particles and other physiochemical properties of dust, such as pH, and OM. The stepwise multiple regression analysis showed that there were significant correlations among pH, OM, the total concentration and the TCLP-extraction (Cd, Pb and Cu) concentration (Table 5). Whereas for As and Cr in street dust, not obvious relationship was found.

**Table 5:** Regression linear analysis of TCLP-extractable contents, total contents and selected dust properties (pH, and OM).

	Regression equation	R	Sig.
As	C = 3.758−0.296pH+0.143OM−0.003Total	0.304	0.044
Cd	C = −0.059+0.004pH+0.001OM+0.197Total	0.547	0
Cr	C = 9.340−0.069pH+1.402OM+0.040Total	0.366	0.007
Cu	C = 5.310−0.300pH+1.678OM+0.053Total	0.660	0.000
Pb	C = 7.939−0.786pH+2.582OM+0.019Total	0.622	0

### Metal bioaccessibility and relationship with dust properties

Many factors can affect metal bioaccessibility in dust, such as the interactions of metals, properties and constituents of dust. The range of bioaccessibility of metals varied widely among urban street dust, As is 4.94% to 37.60%, Pb is 4.49% to 33.22%, Cd is 8.81% to 43.13%, Cr is 6.70% to 80.70% and Cu is 4.21% to 36.92%. The sequence of average bioaccessibility of metals is Cr (22.63%) > Cd (21.73%) > Cu (19.01%) > Pb (15.04%) > As (14.42%). The bioaccessibility of metals (Pb 59%, and Cu 58%) in Hong Kong urban soils (pH 6.6, SOM 4.4%, clay 7%, and sand 76%) [16] was higher, those (As 27.3%, Pb 71.7%, Cr 5.6%, and Cu 40.4%) almost all higher except Cr in urban roadside soils from Xuzhou, China [44].

Metal bioavailability could be affected by the ingested heavy metal-particles and other physiochemical properties of dust, such as pH, and OM. The stepwise multiple regression analysis showed that there were significant correlation relationships among pH, OM, the total metal concentration and the SBET-extraction (As, Pb, Cd, and Cu) concentration (Table 6). Whereas for Cr in street dust, no obvious relationship was found.

**Table 6:** Regression linear analysis of SBET-extractable contents, total concentrations and selected dust properties (pH, and OM).

	Regression equation	R	Sig.
As	C = −0.338+1.000pH+0.366OM+0.015Total	0.506	0
Cd	C = 0.071−0.009pH+0.020OM+0.131Total	0.692	0
Cr	C = 9.340−0.069pH+1.402OM+0.040Total	0.366	0.007
Cu	C = 5.310−0.300pH+1.678OM+0.053Total	0.660	0.000
Pb	C = 7.939−0.786pH+2.582OM+0.019Total	0.622	0

### Ecological risk assessment based on *RI* and *P*

To get better image of urban street dust pollution and related risks, the Hakanson's method and the Nemerow's method were applied in this study. The ecological risk assessment results of metals in urban street dust were summarized in Table 7. It was found that the sequence of risk indices (*E_i_*) of single metal was As > Cd > Pb > Cu > Cr. As and Cd are considerable contaminated in urban street dust, the values of *E_i_* were higher than 100, and less than 160. Pb, Cu and Cr were low contaminated, the values of *E_i_* were lower than 40. According to the character of metals, the overall potential ecological risk of the observed metals was quantified. *RI* was calculated as the sum of all the five risk factors. *RI* in urban street dust was 283.72, which mean that the metals in urban street dust were contaminated in the moderate degree (150≤*RI*<300). The sequence of *P* index of each toxic metal was As > Pb > Cd > Cr > Cu (Table 7) in urban street dust. The ecological risk of As and Pb was low, whose value exceeded 1.0, and was less than 2.0. The ecological risk of Cd, Cu and Cr was barely not polluted, whose value was lower than 0.7. From the results, the potential ecological risk of the toxic metals in the extraction of TCLP was lower than that in the total concentrations, and As was the major contaminant in street dust.

**Table 7:** The potential ecological risk indexes.

Contaminant	*E_i_*	Risk degree	*RI*	*P*	Risk degree
As	108.81±7.18	Considerable	283.72 Moderate degree	1.40	Low
Pb	15.03±0.67	Low		1.36	Low
Cd	148.54±6.59	Considerable		0.51	Clean
Cu	9.63±0.48	Low		0.16	Clean
Cr	1.71±0.08	Low		0.40	Clean

### Human health risk assessment according to oral bioavailability

Metals are usually non-degradable in the environment, and homeostasis mechanisms are unknown. Thus, biological life would be threaten by any high levels of metals [45]. In an individual lifetime, the incremental risk probability of carcinogens is estimated as a result of exposure to the potential carcinogens. In this study, only As and Cr were assessed through the ingestion exposure modes of urban street dust, for children, As was 1.50×10^−3^, and Cr was 5.12×10^−4^, compared to adults, As was 7.49×10^−4^, and Cr was 2.56×10^−4^. The total cancer risk for children was 2.01×10^−3^, and that for adults was 1.05×10^−3^. The cancer risk was higher than 1×10^−4^, which was considered to be high potential risk and indicated that the carcinogenic risk of As and Cr in urban street dust exposure cannot be tolerable.

Non carcinogenic risks of a metal in urban street dust is potential, and non-carcinogenic toxicity can occur with time, which is not expressed as the probability of an individual metal with an adverse effect. In this study, the HQ sequence of toxic metals was As > Cr > Pb > Cu > Cd (4.56×10^−1^, 6.60×10^−2^, 3.25×10^−2^, 3.22×10^−3^ and 1.33×10^−3^, respectively, for children; and 2.28×10^−1^, 3.30×10^−2^, 1.63×10^−2^, 1.61×10^−3^ and 6.67×10^−4^, respectively, for adults) (Table 4). So the *HQ* values for toxic metals in this study were all lower than 1.0, which means that it was at the safe level. Non-carcinogenic risks were safe for children and adults. The hazards index (*HI*) for toxic metals to residents through the daily ingestion of street dust was 5.88×10^−1^ for children, and 2.80×10^−1^ for adults. *HI* was less than 1.0, which means that human exposure to urban street dust was safe.

## Discussion

Street dust pollution in urban areas has an important impact on the environment, human health and life quality of residents. Meanwhile, the health of children and adults was affected by inhaling or ingesting street dust with the high metal contamination. It is important to identify the origin and distribution of toxic metals in street dust. The concentration of As in street dust was at the highest level, then followed by Cr, Pb, Cu and Cd. In most urban areas, it contaminated with As. Only the urban areas close to stations, commercial zones and construction fields, it contaminated with Cd and Cu. Compared with the average concentration of metals in street dust from different cities (Table 4), the average concentration of Pb, Cd, Cr and Cu in Tianjin was nearly same as that in Beijing, but lower than that in Shanghai [46], and the average concentration of Pb, Cu and Cd was almost at the same level in Shenyang [47].

Tianjin has a high demand on winter heating, from November to next March. As is known to be serious concentrated in coals, and coal combustion released many metals, such as As, Cr, Pb and Cu. Particularly, vehicle emissions are a major contributor to other metals in urban street dust, including Pb, Cu and Cd. Vehicle loadings were about 1.76 million in urban areas of Tianjin till 2011. In this study, Cd, Cu and Pb were the main source of auto transport activities, such as vehicle exhaust emissions. Without doubt, there are also a number of other sources of metals in urban street dust, including disintegration of vehicle brakes and tires, atmospheric deposition, road surface wear, municipal solid waste incineration, and residential heating. Besides, many residential buildings are close to streets, which result in the frequent exposure of inhabitants to urban street dust. To reduce the accumulation of toxic metals in street dust, we suggest that it should use clean energy at the heating period and increase green areas in urban areas.

The results of the potential ecological risk for urban street dust using Hakason index and Nemerow index methods were different. The *RI* values of As, Cd, Cu and Cr were higher than *P*, only the risks of Pb were the same. From the results, *RI* could characterize the sensitivity of the toxic metals, and moreover, represent ecological risk resulted from the all contamination in local ecosystems. *RI* estimated the risk in the chemical forms of toxic metals. The categories, concentrations and toxic characteristics of metals could affect the values of *E_i_* and *RI*, and cause the different results. The *P* index was used to estimate the mobility of toxic metals in urban street dust. As and Pb were the major contaminants for the mobility and toxicity, then followed by Cd, Cr and Cu. Despite the concentration of Pb was not higher than Class III of the Chinese National Soil Standards, the *P* index of Pb was still at the low risk due to the low concentrations of Pb in most urban areas. The results of assessment of chemical forms and mobility of metals were different, because the mobility of metals could be reduced by organic matters in dust samples. In other words, the chemical forms and mobility of metals should be coupled in order to get better assessment of their potential ecological risk. Meanwhile, the properties of urban street dust also affect the mobility and bioaccessiblity of metals in street dust. Toxic metals such as As, Pb, Cd, Cr and Cu could continuously accumulate in urban environment due to their non-biodegradability and the longer residence time. Thus, the local government should pay more attention to the action of reckless and unconscious pollution of the environment, and work out some particular management strategies to achieve better urban environmental quality.

Metals may accumulate in fatty tissues of human bodies, have middle and long-term health risks, and can adversely affect their physiological functions, disrupt the normal functioning of internal organs, or act as cofactors in other diseases [48], [49]. Meanwhile, metals could deposite in the circulatory system of people, and each element (As, Cr, Pb, Cu, or Cd) has a distinctive toxicological picture and a particular distribution in human bodies. For instance, Pb accumulates primarily in the liver and in the kidney, while Cd concentrates in the kidney. All these elements originate, following a chronic exposure, complex systemic alterations so that it seems to be quite short-sighted to reduce their toxicological effects to the central nervous system and the cardiovascular system. Metals in urban soils might be transferred to human bodies via ingestion, dermal contact, or breathing, especially to children due to the “hand to mouth” activity during outdoor activities in playgroud and recreational areas [50], [51]. The total carcinogens risk was 2.01×10^−3^ for children, and 1.05×10^−3^ for adults. In other words, they were both higher than 1×10^−4^. With the results, we considered to be high potential risk and indicated that carcinogenic risk of As and Cr in urban street dust exposure cannot be tolerable. *HI* for toxic metals were 5.88×10^−1^ for children, and 2.80×10^−1^ for adults, which means that exposure to urban street dust might be safe. Despite *HI* for children could be safe, low tolerance to toxins and the ingestion of dust through hand-to-mouth pathways, the hazards to children cannot be ignored. The ingestion of dust appears to be the main exposure to street dust, which results in health risk for As, Pb, Cd, Cr and Cu in street dust from anthropogenic sources. But if exposure frequency or ingestion rate is increased, street dust can still result in non-carcinogenic adverse effects on children. Therefore, the potential health risk for children cannot be ignored due to the exposure to the street dust.

Urban street dust receipt large amounts of metals originating from a variety of sources including materials, industrial waste, vehicle emissions, coal burning and other anthropogenic activities. Many metals could be remained in urban street dust for a long time, which may lead to further potential threat to ecosystems and human health. It is well known that chemical speciation of metals should be considered in addition to total concentration when evaluating the potential risk of metals in urban street dust. Therefore, we should consider the mobility and bioavailability of metals in urban street dust in order to offer an insight into potential ecological risk of urban street dust to the environment and human health.

### Statement

Our sampling sites belong to local government departments, do not involve the use of private land. The whole sampling was finished in the public land, did not involve the destruction of the vegetation and biological species.
